# Great American Smokeout — November 15, 2018

**DOI:** 10.15585/mmwr.mm6744a1

**Published:** 2018-11-09

The American Cancer Society’s 43rd annual Great American Smokeout will be held on November 15, 2018. The Great American Smokeout is an annual event that encourages smokers to make a plan to quit smoking (https://www.cancer.org/healthy/stay-away-from-tobacco/great-american-smokeout.html).

A report in this issue of MMWR (*1*) indicates that in 2017, 14.0% of U.S. adults were current cigarette smokers, the lowest prevalence recorded since monitoring began in 1965. Nonetheless, smoking remains the leading preventable cause of disease, disability, and death in the United States (*2*). Each year, an estimated 480,000 U.S. adults die from cigarette smoking and secondhand smoke exposure (*2*).

Smokers can and do quit smoking: former smokers now outnumber current smokers (*2*). Among current U.S. adult smokers, nearly two out of three want to quit smoking, and approximately half made a quit attempt in the preceding year (*2*). Getting effective help through counseling and use of medications can increase the chances of quitting by as much as threefold (*3*).

Information and support for quitting smoking is available at 800-QUIT-NOW (800–784–8669). CDC’s Tips From Former Smokers campaign offers additional resources (https://www.cdc.gov/tips).
